# Lrp4 Regulates Initiation of Ureteric Budding and Is Crucial for Kidney Formation – A Mouse Model for Cenani-Lenz Syndrome

**DOI:** 10.1371/journal.pone.0010418

**Published:** 2010-04-29

**Authors:** Courtney M. Karner, Martin F. Dietrich, Eric B. Johnson, Natalie Kappesser, Christian Tennert, Ferda Percin, Bernd Wollnik, Thomas J. Carroll, Joachim Herz

**Affiliations:** 1 Department of Internal Medicine, University of Texas Southwestern Medical Center, Dallas, Texas, United States of America; 2 Department of Molecular Genetics, University of Texas Southwestern Medical Center, Dallas, Texas, United States of America; 3 Department of Medical Genetics, Faculty of Medicine, Gazi University, Ankara, Turkey; 4 Center for Molecular Medicine Cologne (CMMC) and Institute of Human Genetics, University of Cologne, Cologne Excellence Cluster on Cellular Stress Responses in Aging-Associated Diseases (CECAD), Cologne, Germany; University of Giessen Lung Center, Germany

## Abstract

**Background:**

Development of the kidney is initiated when the ureteric bud (UB) branches from the Wolffian duct and invades the overlying metanephric mesenchyme (MM) triggering the mesenchymal/epithelial interactions that are the basis of organ formation. Multiple signaling pathways must be integrated to ensure proper timing and location of the ureteric bud formation.

**Methods and Principal Findings:**

We have used gene targeting to create an Lrp4 null mouse line. The mutation results in early embryonic lethality with a subpenetrant phenotype of kidney agenesis. Ureteric budding is delayed with a failure to stimulate the metanephric mesenchyme in a timely manner, resulting in failure of cellular differentiation and resulting absence of kidney formation in the mouse as well as comparable malformations in humans with Cenani-Lenz syndrome.

**Conclusion:**

Lrp4 is a multi-functional receptor implicated in the regulation of several molecular pathways, including Wnt and Bmp signaling. Lrp4^−/−^ mice show a delay in ureteric bud formation that results in unilateral or bilateral kidney agenesis. These data indicate that Lrp4 is a critical regulator of UB branching and lack of Lrp4 results in congenital kidney malformations in humans and mice.

## Introduction

The definitive kidney forms as a result of inductive interactions between the metanephric mesenchyme and the UB [1]. In the mouse, signals from the metanephric mesenchyme stimulate the ureteric bud to branch from the Wolffian duct around embryonic stage E10.5 [2]. The UB subsequently invades the overlying metanephric mesenchyme and produces signals that are necessary for survival, proliferation and differentiation of the mesenchyme [3]. The timing and location of ureteric budding are critical factors in kidney organogenesis. Genetic and surgical manipulations have revealed that the mesenchyme is only competent to respond to signals from the bud for a narrow time window [4]. Failure of the bud to reach the mesenchyme in this narrow window results in apoptosis of the mesenchyme and subsequent kidney agenesis [5], [6]. Defects in secondary branching of the ureteric bud can result in a range of phenotypes ranging from congenital anomalies like hypoplastic kidneys to cystic dysplasia [7].

Defects in kidney formation constitute some of the most common birth defects in humans [8]. Multiple signaling pathways have been implicated in UB branching. The GDNF/Ret, FGF and Wnt signaling pathways are necessary for normal branching [4], [9], [10] while the BMP pathway appears to act as a branching inhibitor [11]. As would be expected, tight regulation of these pathways is essential to insure the proper timing and location of branching. Although we have gained a great deal of information on the molecular mechanism regulating ureteric bud branching in mice, there has been surprisingly little correlation between these major pathways and congenital defects in man [12].

Lrp4 is a member of the low-density lipoprotein (LDL) gene family [13]. Mutations in this membrane receptor have been implicated in neuromuscular junction [14], limb and tooth development where it appears to integrate signaling from multiple pathways including Wnts and Bmps [15], [16], [17]. Here, we describe an additional role for Lrp4 in the formation of the UB. Loss of Lrp4 results in a delay in UB formation and a subpenetrant kidney agenesis phenotype. We also identified mutations in Lrp4 in humans with congenital kidney defects [18]. These studies establish Lrp4 as a critical regulator of ureteric budding in both mice and humans.

## Materials and Methods

### Mouse Strains

*HoxB7-Cre* and Catnb^exon3flox^ mouse lines have previously been described [19], [20]. The Lrp4 knockout (KO) mouse was generated by replacing the first exon with a neomycin resistance cassette using techniques described previously [15]. The long arm of homology upstream of the first exon of Lrp4 was generated by PCR using primers MEJ23 (5′-GCGGCCGCCAGGTCATGAAGTGAGTGCTGAGCCACTGGG-3′) and MEJ24 (5′-CCACCACCGCCTCATGGTGCTGCGGCCGCC-3′). The short arm of homology downstream of the first exon of Lrp4 was generated by PCR amplification using the primers MEJ33 (5′-CTCGAGGAGCGGTCTGCAGATCCTGGCGATTCACGG-3′) and MEJ35 (5′-CTCGAGGGTTACAGACTCTGCAACTGCTCTACCTCATTG-3′). The long arm and short arm of homology were cloned into pJB1 using the NotI and XhoI restriction sites, respectively.

Mice were maintained on a mixed 129/C57 background. All animal work was conducted according to the relevant national and international guidelines and in accordance with the recommendations of the Weatherall report, “The use of non-human primates in research” (no primates were used in this study). Animal experiments conducted in Dallas were also reviewed and approved by the Institutional Committee on Animal Use and Care (IACUC) at UT Southwestern Medical Center.

### Genotyping

KO Mice were genotyped by PCR as follows: MEJ358 (5′-ACTATATTCACCCGCCGGCTTTTCCACGTG-3′) and KOT12 (5′-AGCAGCTTTCAGAAGCACCTCTTCAGGACC-3′) were used to selectively amplify the wild-type allele and Neo36 (5′-CAGGACAGCAAGGGGGAGGATTGGGAAGAC -3′) and KOT12 were used to amplify the knockout allele. The HoxB7Cre allele was amplified using the primers 5′-CCATGAGTGAACGAACCTGG-3′ and TGATGAGGTTCGCAAGAACC to give a 400 base pair band using the conditions previously described. The β-catenin exon3flox allele was amplified using the primers: 5′-AACTGGCTTTTGGTGTCGGG-3′ and 5′-TCGGTGGCTTGCTGATTATTTC-3′. Using a 55°C extension temperature, the wild type allele yields a 291 base pair band while the exon 3 floxed allele yields a 400 base pair band.

### *In situ* hybridization

Whole-mount *in situ* hybridization was performed as previously described using the following antisense probes: cRet, Wnt11, Pax2, Wnt9b, Lrp4 and GDNF [15], [19]. Briefly, embryos were harvested and fixed in 4% paraformaldehyde in PBS at 4°C overnight. Embryos were treated with 10 µg/ml proteinase K in PBST for 20 minutes at room temperature and hybridized overnight at 72°C with digoxigenin-UTP labeled probes. Embryos were then incubated overnight at 4°C with alkaline phosphatase-coupled anti-digoxigenin antibody (Roche Applied Science). Color reaction was developed using BM Purple (Roche).

### H&E histology

Kidneys from P0 pups were emersion fixed with 10% formalin and embedded in paraffin. The kidneys were then sectioned and stained with H&E using standard techniques.

### Whole mount antibody staining

Embryonic day 10.5 embryos were dissected in PBS and staged according to somite number. Embryos at the 38 somite stage were fixed overnight in 4% PBS (Electron microscopy services) overnight at 4°C. After fixation embryos were dehydrated and rehydrated through a graded ethanol series. Embryos were then washed four times for 30 minutes at room temperature with heavy agitation in PBS +0.1% Triton-X (PBStx). Embryos were blocked for at least 3 hours at room temperature in 10% FBS/PBStx. Embryos were incubated with antibodies to E-Cadherin (Rat 1∶400 Zymed) and Pax2 (Rabbit 1∶400 Covance) overnight at 4 degrees Celsius, then washed six times 30 minutes each wash at room temperature in PBStx. Embryos were incubated with fluorescently coupled secondary antibodies (Molecular probes) overnight at 4°C followed by extensive washing in PBStx. Wolffian ducts were then dissected away from the embryo and imaged on a Zeiss NeoLumar stereoscope using an Olympus DP71 camera.

## Results

### Lrp4 is required for kidney formation

**Figure 1:**
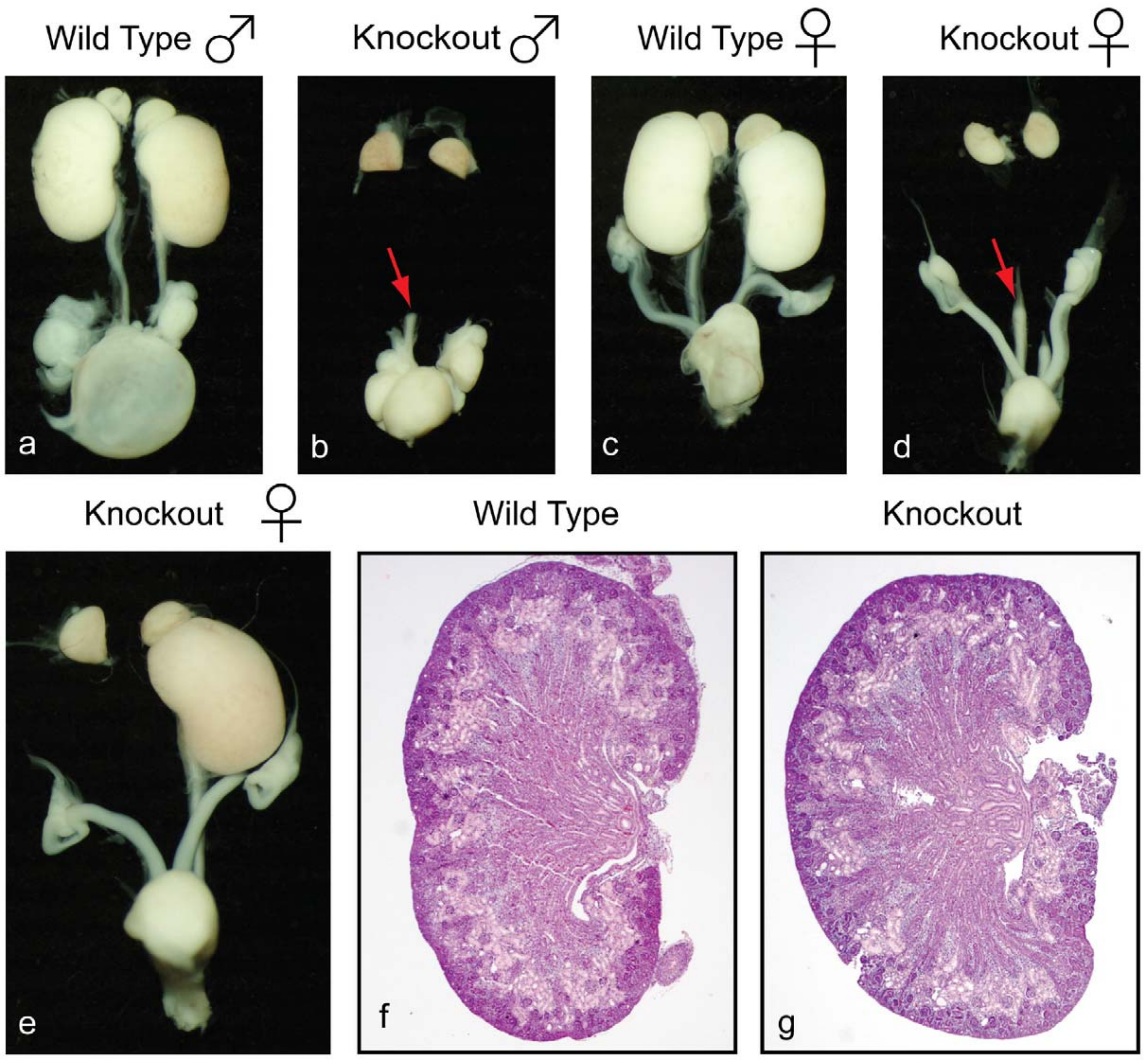
Unilateral and bilateral kidney agenesis in LRP4 knockout mice. Kidney agenesis in the Lrp4 knockout (b,d,e). Bilateral (b,d) or unilateral (e) kidney agenesis with rudimentary ureters (red arrows). The lower urinary and genital systems of males and females remain intact. Histological analysis (Hematoxylin-Eosin stain) does not reveal morphological defects in the kidneys that form in Lrp4 knockout animals (g) compared to the wild-type kidneys (f).

We have generated mice that harbor a null allele of Lrp4 by replacing exon1 with a neomycin stop cassette (Lrp4^−/−^). In the examination of post-partum Lrp4^−/−^ mice (n = 156) we found 51 percent bilateral and 22 percent unilateral kidney agenesis (Fig. 1b, d, e). This distribution was gender-independent and involved only structures derived from the UB and metanephric mesenchyme (MM) (Fig. 1a–d). The small number of kidneys that did form in Lrp4 knockouts were indistinguishable from wild-type at both the histological and molecular level (Fig. 1f and g). Functional analysis was not possible due to the immediate post-partum lethality caused by neuromuscular junction defects [14].

### Lrp4 is widely expressed in the kidney during development

**Figure 2:**
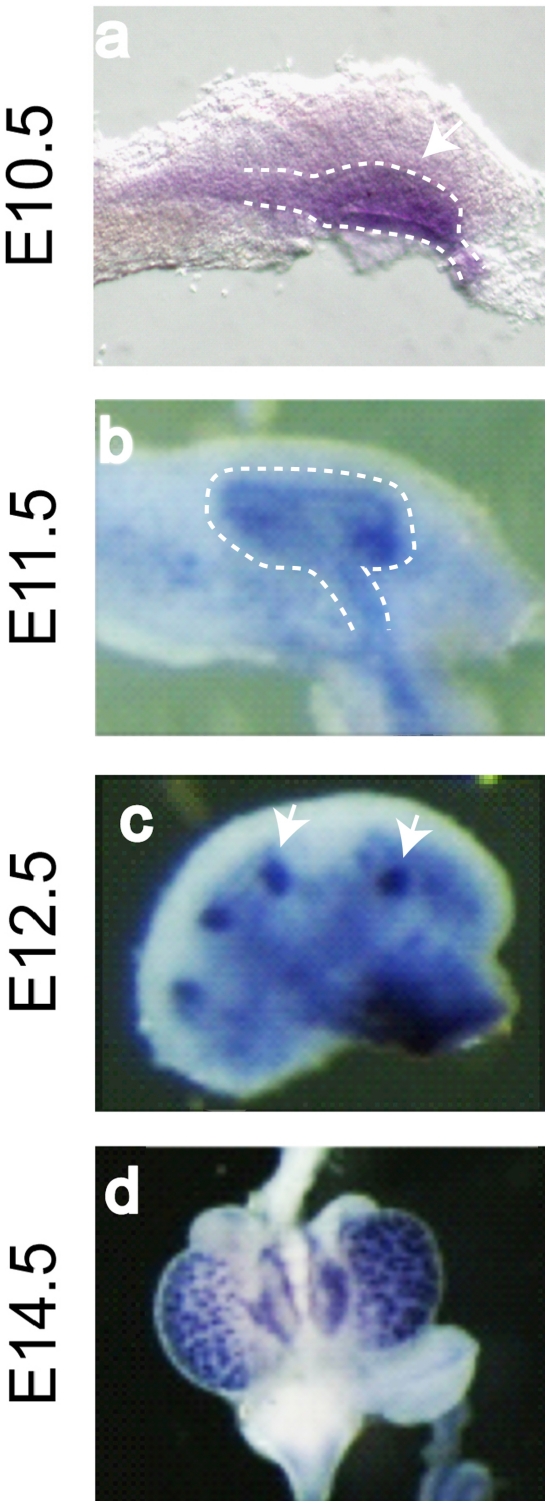
Expression of Lrp4 in the developing kidney. At E10.5 Lrp4 is expressed throughout the Wolffian duct and the ureteric bud. (a). At E11.5, Lrp4 is expressed in the ureteric bud and the pre-tubular aggregates (b). At E12.5 and E 14.5, Lrp4 expression is maintained in the ureteric bud and the renal vesicles (c and d, respectively). The Wolffian duct and ureteric bud are outlined by dotted lines; the arrow points to the early ureteric bud in (a) or the renal vesicles in (c), respectively.

To better understand its contribution to kidney formation, we investigated the expression of Lrp4 during development. Beginning at the initiation of kidney development, embryonic day (E) 10.5, Lrp4 mRNA is visible in the mesonephric tubules and the Wolffian duct adjacent to the MM (Fig. 2a). At E11.5, Lrp4 is expressed throughout the ureteric epithelium and the adjacent pre-tubular aggregates (Fig. 2b). Lrp4 continues to be expressed in the ureteric bud derived epithelia and the pre-tubular aggregates/renal vesicles throughout the embryonic period (Fig. 2a–d).

### Pax2 signaling remains intact in the absence of Lrp4

**Figure 3:**
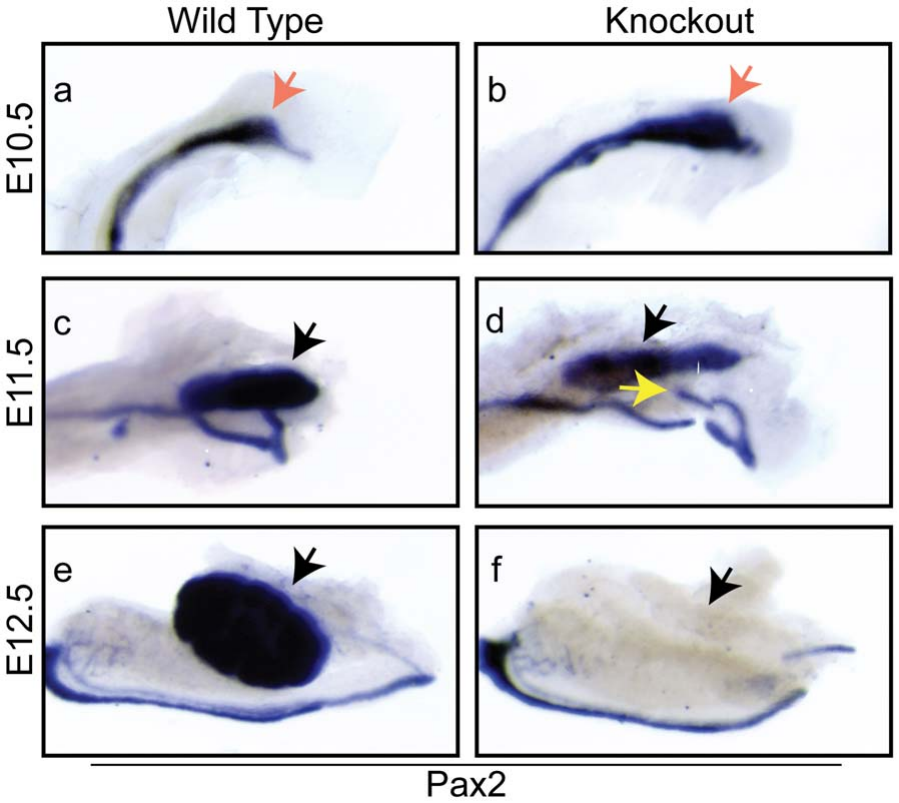
Pax2 signaling remains intact in the absence of Lrp4. Pax2 is expressed normally in the metanephric mesenchyme and the ureteric bud, indicated by the red arrows, at E10.5 in the wild type and Lrp4 knockout mice (a and b). At E11.5, Pax2 is expressed normally in both the ureteric bud and metanephric mesenchyme, the latter indicated by black arrows, of wild type (c) and Lrp4 knockout animals (d). However, the ureteric bud fails to invade the metanephric mesenchyme and does not undergo secondary branching in the Lrp4 knockout indicated by the yellow arrow (d). The black arrows indicate mesenchymal expression of Pax2, which is present in the wild-type, but subsequently lost in the knock-out kidney mesenchyme at E12.5 (e and f).

To gain insight into the nature of the mutant defect, we evaluated the expression of a series of genes necessary for kidney development. Pax2 is a critical regulator of kidney branching that is normally expressed in the Wolffian duct, the ureteric bud/collecting ducts and the metanephric mesenchyme throughout the developmental period [21], [22], . Pax2 is expressed normally in the Wolffian duct and metanephric mesenchyme in both wild type and Lrp4 mutants through E11.5 (Fig. 3a–d), although at E11.5 the mutant ureteric bud has not contacted the mesenchyme and has not formed a T-shape (Fig. 3d). Failure of the ureteric bud to invade the metanephric mesenchyme leads to a loss of the metanephric mesenchyme and kidney agenesis [6]. In support of this hypothesis the mesenchymal expression of Pax2 is lost by E12.5 in Lrp4^−/−^ animals (Fig. 3f).

### The GDNF/Ret/Wnt11 signaling network is unaffected by Lrp4

**Figure 4:**
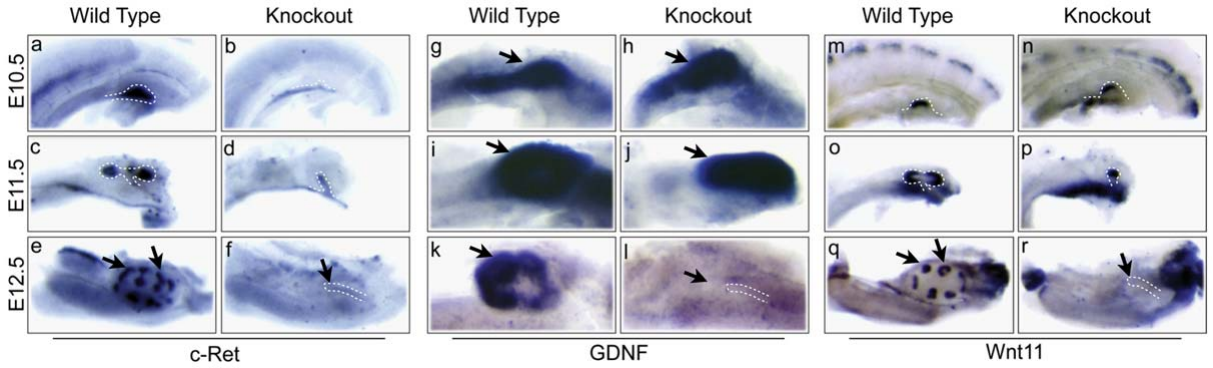
Expression of branching regulators in Lrp4 mutants. Expression of c-Ret (a–f), GDNF (g–l) and Wnt11 (m–r) in E10.5 (a,b,g,h,m and n), E11.5 (c,d,i,j,o and p), and E12.5 (e,f,k,l,q,r) in wild-type (a,c,e,g,i,k,m,o, and q) and Lrp4 knockout (b,d,f,h,j,l,n,p and r) kidneys. C-Ret is expressed in the ureteric bud at basal levels in the Lrp4 knockout mice at E10.5 (a,b). At E11.5, the Lrp4 knockout ureteric bud fails to bifurcate or upregulate c-Ret expression at the tip of the ureteric bud (d) compared to wild-type embryos (c). At E12.5, the signal is greatly reduced in the knockout kidney (f). GDNF is expressed normally in the metanephric mesenchyme at both E10.5 and 11.5 in wild type and Lrp4 knockout animals (g–j). By E12.5, GDNF expression is completely lost from the Lrp4 knockout metanephric mesenchyme (k and l). Wnt11 is expressed normally at the tips of the ureteric bud at both E10.5 (m and n) and 11.5 (o and p) in Lrp4 mutants compared to wildtype. By E12.5 Wnt11 is absent from the ureteric bud of Lrp4 knockout animals (q and r). The Wolffian duct and ureteric bud are outlined by white dashed lines, mesenchyme (g, h, I, j) renal vesicles (e and q) and truncated ureteric bud (f, l, r) are indicated by black arrows.

Kidney development begins at E10.5 when the ureteric bud branches from the Wolffian duct in response to GDNF secreted from the mesenchyme. The apparent failure of the ureteric bud to reach the mesenchyme in Lrp4 mutant kidneys is similar to what has been observed in mice with defects in GDNF/Ret signaling [24]. GDNF is a ligand for c-Ret and a co-receptor, GFRα1 [11]. Mutations in any of these three genes results in partially penetrant kidney agenesis. To examine potential defects in the GDNF/Ret pathway, we first examined the expression of Ret and GDNF mRNA. At E10.5, c-Ret is expressed in the ureteric bud at equivalent levels in the Lrp4 knockout mice compared to their wild type counterparts (Fig. 4a and b). At E11.5 the ureteric bud has invaded the mesenchyme, bifurcated and upregulated c-Ret at the ureteric tips while no bifurcation or upregulation of c-Ret occurs in Lrp4 mutants (Fig. 4c and d). At E12.5, c-Ret levels are greatly reduced in the knockout compared to wild type control (Fig. 4f). As expected, GDNF is expressed in the metanephric mesenchyme at normal levels at E10.5 and E11.5 (Fig. 4g–j). As was seen with Pax2, by E12.5 mesenchymal expression of GDNF is completely lost, consistent with the hypothesis that the ureteric bud has not invaded the metanephric mesenchyme (Fig. 4k and l). We next wanted to test whether Ret/GDNF signaling is intact in Lrp4 mutants. We examined the expression of Wnt11, a GDNF-inducible downstream target of c-Ret [25]. Similar to c-Ret, Wnt11 expression is upregulated in the tips of the bud at E10.5 and 11.5. Importantly, Wnt11 is still expressed in Lrp4^−/−^ ureteric buds at E11.5 that have failed to bifurcate, indicating that Ret/GDNF signaling is active despite the apparent failure of mesenchymal invasion. By E12.5 Wnt11 expression is absent, presumably due to the loss of mesenchymal GDNF (Fig. 4a–f).

As the Lrp4 mutant phenotype does not appear to be the result of defects in Ret/GDNF signaling, we examined the activity of other pathways involved in ureteric bud branching. Lrp4 has been implicated in the activity of both Bmp [26], [27] and Wnt [28], [29] signaling and both of these pathways play roles in normal branching morphogenesis. To test for defects in Bmp signaling, we investigated the expression of phosphorylated Smads1, 4 and 8. We were unable to detect differences in either the level or location of p-Smad staining in either the mesenchyme or ureteric buds of Lrp4 mutants at either E10.5 or 11.5 (data not shown). To assay Wnt signaling, we examined the expression of Axin2 mRNA in the Wolffian duct and ureteric bud. Similar to the situation with the p-Smads, we were unable to detect significant differences in transcript levels (data not shown).

### Ureteric budding is delayed in Lrp4 null mice

**Figure 5:**
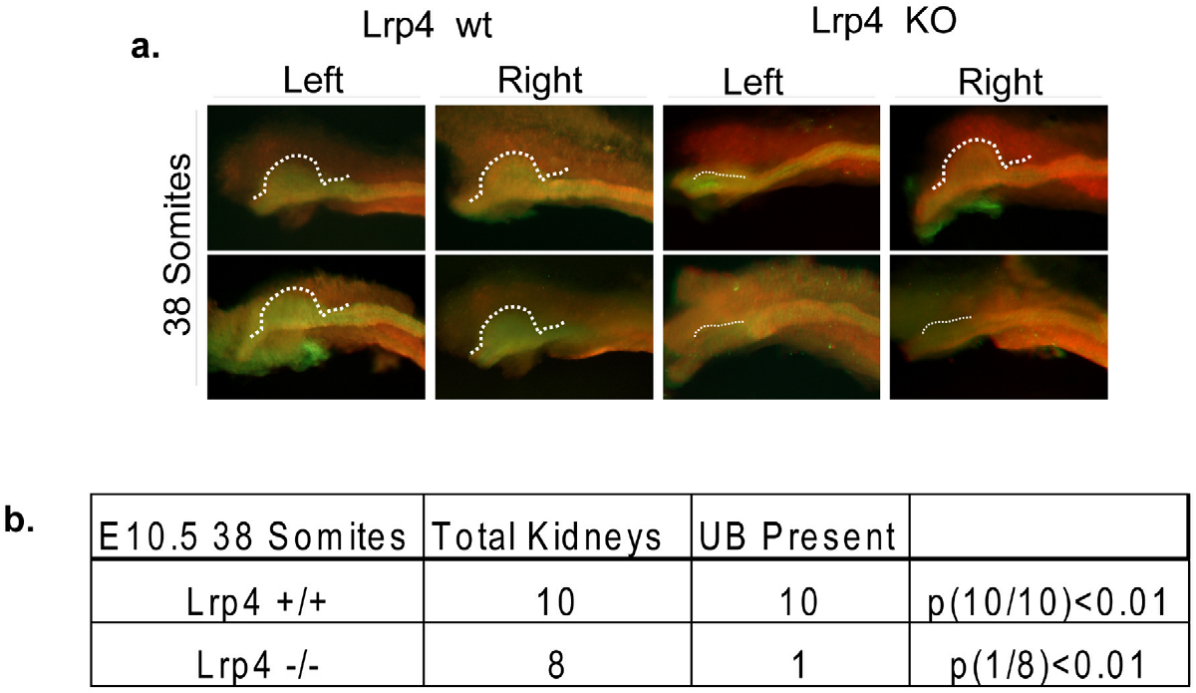
Ureteric Budding is delayed in Lrp4 Mutants. 38 somite stage E10.5 embryos were stained with the epithelial markers Pax2 (red) and E-cadherin (green) to label the Wolffian duct and developing ureteric bud. Representative images of kidney pairs for two wild-type and knock-out animals are shown. In the wild-type (a–d), ureteric buds appear as expected while there is a frequent delay in ureteric bud outgrowth in the Lrp4 mutants (e,g,h). One Lrp4 mutant animal is shown with a unilateral outgrowth (f). In total, all 10 expected buds are formed at the 38 somite stage in the wild-type background while only 1 out of 8 predicted buds is present in the knock-out (Panel b). P values (Student's t-test) p<0.01 indicates significance.

The absence of metanephric mesenchyme at E12.5 is indicative of a failure of the UB to reach these cells and provide survival signals. The expression of Pax2 (Fig. 3d) indicated that the UB bud is delayed in reaching the mesenchyme, which could be due either to defects in growth of the ureteric bud or a delay in formation of the bud. The complete lack of a phenotype in some mutants seemed more in line with a delay in bud invasion. To investigate the possibility of a budding delay as a possible explanation of our phenotype, we examined ureteric bud formation at E10.5. Stage and somite matched embryos were stained for the epithelial markers Pax2 and E-cadherin to assess UB formation. E-cadherin is a marker for the epithelial structures of the ureteric bud; Pax2 is expressed in both epithelium and mesenchyme alike. We proceeded with double-staining for a clear orientation within the slide. Interestingly, although we noticed at least a partial ureter in all newborn Lrp4 mutants, we found that the UB had formed in only 12.5% (1/8) of 38 somite stage Lrp4 mutants (compared to 100% of cases for wild type controls) (Fig. 5a and b). These data indicate that ureteric bud formation is delayed in mutants, and that the failure to invade the mesenchyme in time to support normal growth/survival is the cause for the frequent uni- or bilateral kidney agenesis.

### Wnt overexpression in the ureteric bud leads to kidney agenesis

**Figure 6:**
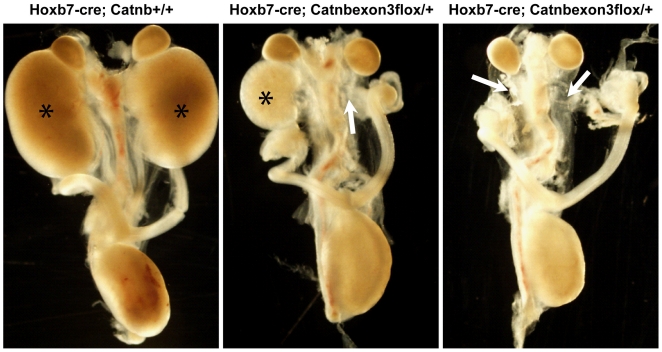
Wnt Overexpression in the Ureteric Bud Leads to Kidney Agenesis. Expression of a stabilized allele of β-catenin (Catnb^exon3flox^) in the Wollfian duct using HoxB7Cre to activate transgene expression phenocopies the Lrp4 knockout phenotype with both uni- and bilateral kidney agenesis (a-c). The formation of the Wolffian duct and distal ureters as well as bladder and adrenal glands remained unaffected. The asterisks (a and b) indicate the position of regular kidneys. The arrows (b and c) indicate the predicted position of kidneys that have not formed.

Lrp4 is a negative regulator of the Wnt signaling pathway. This lead us to hypothesize that overactive Wnt signaling in mutants could be responsible for the delay in ureteric bud formation. We therefore tested whether expression of a constitutively active β-catenin transgene would result in a comparable phenotype to the absence of Lrp4. Expression of this transgene under the control of a HoxB7Cre promoter, which is restricted to the ureteric bud epithelium indeed resulted in a comparable kidney agenesis phenotype (Fig. 6a–c). The formation of the Wolffian duct and distal ureters as well as bladder and adrenal glands remained unaffected. The similarity of these two distinct animal models suggests a role for deregulated Wnt signaling in the generation of the Lrp4 knockout phenotype.

### Lrp4 binds Gremlin1, a positive regulator of ureteric budding

**Figure 7:**
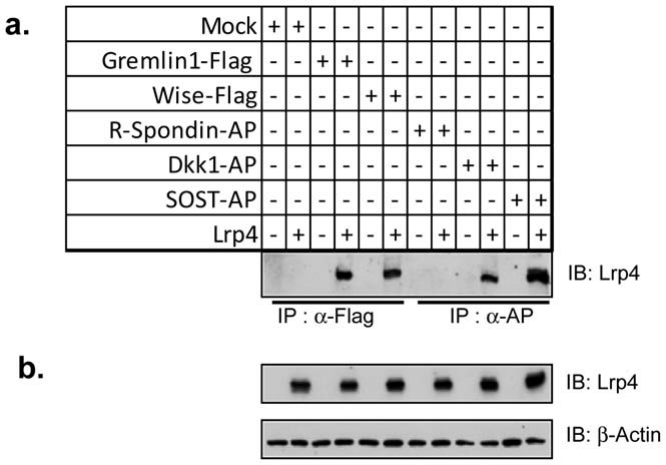
Lrp4 binds the Bmp4 antagonist Gremlin1 *in vitro*. Lrp4 has been implicated in modulating the Bmp signaling pathway through binding of the Wnt and Bmp modulator Wise. Co-immunoprecipitation reveals Gremlin1 binding to Lrp4 *in vitro* (Panel A lane 4); we further confirmed the Lrp4 binding partners Wise, Dkk1 and SOST (Panel A, lanes 6, 10 and 12). The Wnt agonist R-spondin 2 did not interact with Lrp4 (Panel A lane 7 and 8). Transfection efficiency was confirmed by immunoblot analysis (Panel B).

Lrp4 has been established as a regulator of both the Wnt and Bmp signaling pathways. This involves, at least in part, the binding of signal modulating ligands to the extracellular domain. We tested Gremlin1, a facilitator of ureteric budding, as a possible candidate. Previously, Gremlin1 has been reported to antagonize Bmp4 signaling and its deletion in mice results in a renal phenotype with skeletal involvement similar to the Lrp4 knockout. In co-immunoprecipitation experiments, Gremlin1 binds to Lrp4 (Fig. 7). Although we failed to detect a direct difference in Bmp pathway activation at the protein level, Lrp4 might function by facilitating the presentation or integration of Gremlin1 into a signaling complex that mediates the activation of ureteric budding.

### Lrp4 mutations cause renal malformations in humans

**Figure 8:**
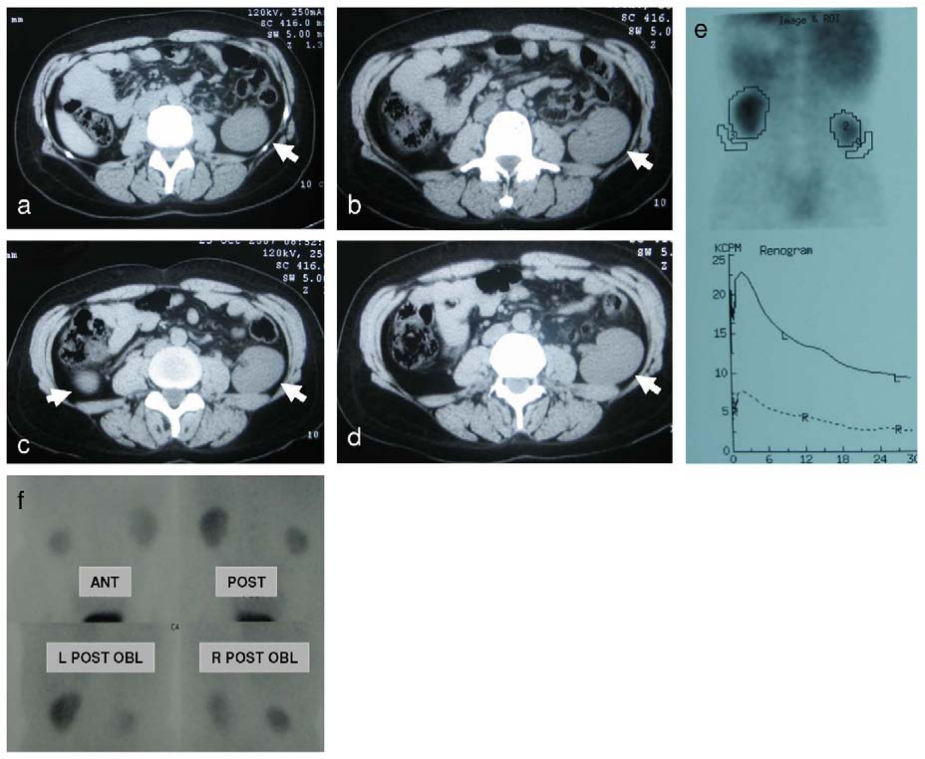
Hypoplastic and Hypofunctional Kidney in Human Lrp4 Mutations. CT scan reveals a severely hypoplastic kidney on the right and mild hypoplasia on the left side (a–d). Both kidneys are ectopic with caudal and lateral shifts (a–d). Dynamic-static renal scintigraphy with Tc-99m DTPA suggest right kidney dysfunction (e). Global renal functional participation; right kidney 26% and left kidney 74%. Static renal cortical scintigraphy with Tc-99m DMSA background activity of radiopharmaceutical is higher than expected (f).

In a cooperative effort, Li et al. [18] identified homozygous LRP4 mutations in patients with Cenani-Lenz syndrome (CLS), a congenital syndrome mainly characterized by musculoskeletal malformations, analogous to the murine phenotype including polysyndactyly and molar fusion. When evaluated for kidney defects, Li and colleagues observed congenital kidney abnormalities in homozygous carriers in more than half of the investigated families, which was hitherto unknown. Renal agenesis, in accordance with the murine phenotype, was observed in one-third of the families, another 25% percent presented with ectopic or hypoplastic kidneys. For one of the affected CLS patients of the CL-6 family described by Li et al. [18], imaging and functional studies revealed ectopic and hypoplastic kidneys on both sides (Fig. 8a–d). Dynamic-static renal scintigraphy with Tc-99m DTPA showed hypofunction of the right kidney, which contributed 26% vs 74% (left kidney) to total renal function (Fig. 8e). Static renal cortical scintigraphy with Tc-99m DMSA revealed increased background activity (Fig. 8f). Creatinine in this patient was elevated at 1.2 mg/dL. Both of these findings indicated impaired renal function. Clinical variability of phenotypic expression suggests that additional modifying factors that affect budding, branching morphogenesis and organ maturation contribute to this phenotype in humans.

## Discussion

In this study, we have shown that Lrp4 functions as a critical regulator of kidney development in both mouse and human. In mice, complete absence of functional Lrp4 leads to uni- or bilateral kidney agenesis caused by a delay in the formation of the ureteric bud. In other mouse models, e.g. the limb deformity (*ld*) mutation or *Danforth's short tail* (*Sd*) mice [30], delayed invasion of the ureteric bud into the receptive mesenchyme results in mesenchymal apoptosis and kidney agenesis [6]. The fact that normal kidneys do develop in a subset of Lrp4 null embryos suggests that the signaling capacity of the bud and the receptivity of the mesenchyme is unaffected by loss of this gene. However, the range of phenotypes observed in humans, from complete agenesis to hypoplasia, along with the expression of Lrp4 mRNA in multiple cell types of the kidney throughout the embryonic period suggest this molecule may have additional roles in kidney development, or that other factors exist, which can modify the phenotype.

The precise mechanism for Lrp4 action during kidney development is still unclear. During kidney development, tissue-tissue interactions between the metanephric mesenchyme and the UB are critical and rely on the integration and regulation of several signaling pathways. Wnt signaling is crucial for UB branching and has been shown to be regulated by Lrp4 in other systems [15], [16], [17]. Intriguingly, a mouse model with UB-specific overexpression of activated β-catenin presents with a very similar phenotype to the Lrp4 mutant (Fig. 6). However, analysis of the Wnt pathway activity has failed to reveal significant changes in Lrp4 mutants, possibly due to high baseline activity in wildtype animals.

An alternative yet equally plausible scenario is that Lrp4 is involved in the modulation of Bmp signaling. We have found that, like other members of the LDL receptor gene family, Lrp4 is capable of modulating TGF-β related signaling [17], [31]. In this study, we have confirmed novel binding partners for Lrp4 including the Bmp regulating ligand Gremlin1 (Fig. 7). As Gremlin1 knockout mice display a phenotype of bilateral kidney agenesis (reportedly due to ectopic Bmp4 activity) [27], an attractive model is that Lrp4 cooperates with Gremlin to inhibit Bmp4 activity. However, similar to the case with β-catenin signaling, we were unable to detect significant changes in the expression of the Bmp targets, pSmad1, 4 and 8. It is therefore possible that Lrp4 acts through an unrelated pathway or perhaps through only partial modulation and integration of both Bmp and Wnt signaling.

Normal kidney formation occurs in a hypomorphic Lrp4 mutant, where only a secreted extracellular domain is expressed, adding additional insight into the mechanism of Lrp4 during ureteric budding [15], [16]. These findings suggest that whatever factor Lrp4 is normally interacting with in the kidney, it is occurring extracellularly and most likely does not require endocytosis of the receptor. Possible mechanisms include quenching of Wnt and BMP modulators, such as Gremlin1 (Fig. 7) by the secreted extracellular domain.

In summary, we have identified Lrp4 as a critical factor for UB outgrowth and kidney formation in the mouse. We have also shown that mutations in Lrp4 lead to the same or very similar developmental malformations as seen in human LRP4 deficient patients with Cenani-Lenz syndrome, further underscoring the importance of Lrp4 for human genetics and medicine.
